# MSMEG_2731, an Uncharacterized Nucleic Acid Binding Protein from *Mycobacterium smegmatis*, Physically Interacts with RPS1

**DOI:** 10.1371/journal.pone.0036666

**Published:** 2012-05-09

**Authors:** Mingzhang Yang, Yuanyuan Chen, Ying Zhou, Liwei Wang, Hongtai Zhang, Li-Jun Bi, Xian-En Zhang

**Affiliations:** 1 Key Laboratory of Non-coding RNA, Institute of Biophysics, Chinese Academy of Sciences, Beijing, China; 2 State Key Laboratory of Virology, Wuhan Institute of Virology, Chinese Academy of Sciences, Wuhan, China; 3 Graduate School, Chinese Academy of Sciences, Beijing, China; University of Queensland, Australia

## Abstract

While the *M. smegmatis* genome has been sequenced, only a small portion of the genes have been characterized experimentally. Here, we purify and characterize MSMEG_2731, a conserved hypothetical alanine and arginine rich *M. smegmatis* protein. Using ultracentrifugation, we show that MSMEG_2731 is a monomer *in vitro*. MSMEG_2731 exists at a steady level throughout the *M. smegmatis* life-cycle. Combining results from pull-down techniques and LS-MS/MS, we show that MSMEG_2731 interacts with ribosomal protein S1. The existence of this interaction was confirmed by co-immunoprecipitation. We also show that MSMEG_2731 can bind ssDNA, dsDNA and RNA *in vitro*. Based on the interactions of MSMEG_2731 with RPS1 and RNA, we propose that MSMEG_2731 is involved in the transcription-translation process *in vivo*.

## Introduction

*Mycobacterium tuberculosis* is the pathogen responsible for human tuberculosis and infects 1–2≥billion people worldwide, causing up to 2≥million deaths annually. There have been significant advances in understanding the biology of this pathogen since the sequencing of the *M. tuberculosis* H37Rv genome was completed in 1998 [1]. However, less than 40% of the genes of this pathogen have a known or a putative function. [1]–[3]. More extensive functional research on its genes, especially mycobacterial specific genes, is required to reveal the mechanisms underlying its growth, pathogenicity and dormancy.

Although the suitability of *M. smegmatis* as a model organism for studying the virulence of *M. tuberculosis* has been questioned, this fast-growing and non-pathogenic species is still of great importance for studying mycobacterial biology [4], [5]. Many genes which were initially described as being *M. tuberculosis*-specific share closely related homologues in *M. smegmatis*. The genome of *M. smegmatis* is 6,988,209≥nucleotides long, approximately 1.7≥times larger than that of *M. tuberculosis*. Nearly a third of the 6716 proteins encoded by the 6938 ORFs identified in the genome of *M. smegmatis* are hypothetical proteins, and only a small portion of the “annotated” genes have been characterized experimentally.

MSMEG_2731 is a conserved hypothetical alanine and arginine rich protein from *M. smegmatis*. MSMEG_2731 contains 454 amino acids and is composed of three tandem repeats of the DUF349 domain [6]. MSMEG_2731 belongs to protein cluster CLSK872100 which contains a total of 18 proteins and is conserved in mycobacteria. Rv2731, the *M. tuberculosis* homologue of MSMEG_2731 shares 73% sequence identity to MSMEG_2731. Rv2731 is present in cytosol, cell wall, and cell membrane fractions of *M. tuberculosis* H37Rv [7], [8]. It was suggested that the expression of *Rv2731*, a conserved hypothetical gene of unknown function made it relevant to investigate whether it plays a role in mycobacterial specific functions [9].

To identify and functionally study this conserved MSMEG_2731, we cloned the *MSMEG_2731* gene and expressed the protein in *E. coli*. After purifying MSMEG_2731 to homogeneity, we identified that MSMEG_2731 mainly exists as monomer *in vitro*. Then we tested the expression of MSMEG_2731 *in vivo* and measured its expression level by quantitative western blot. Protein-protein interactions play a critical role in nearly every aspect of cell structure and function. Identifying protein-protein interactions offers important clues for understanding the function of an unknown protein [10]. Here, using pull-down techniques and LS-MS/MS, we found that ribosomal protein S1 is one of the interaction partners of MSMEG_2731. In addition, we also showed that MSMEG_2731 is a nucleic acid- binding protein which can bind to single stranded DNA, double stranded DNA and RNA *in vitro*. These findings imply that MSMEG_2731 is likely to be a mycobacterial specific factor involved in transcription or translation in *M. smegmatis*.

## Results

### Purification and characterization of recombinant MSMEG_2731

**Figure 1:**
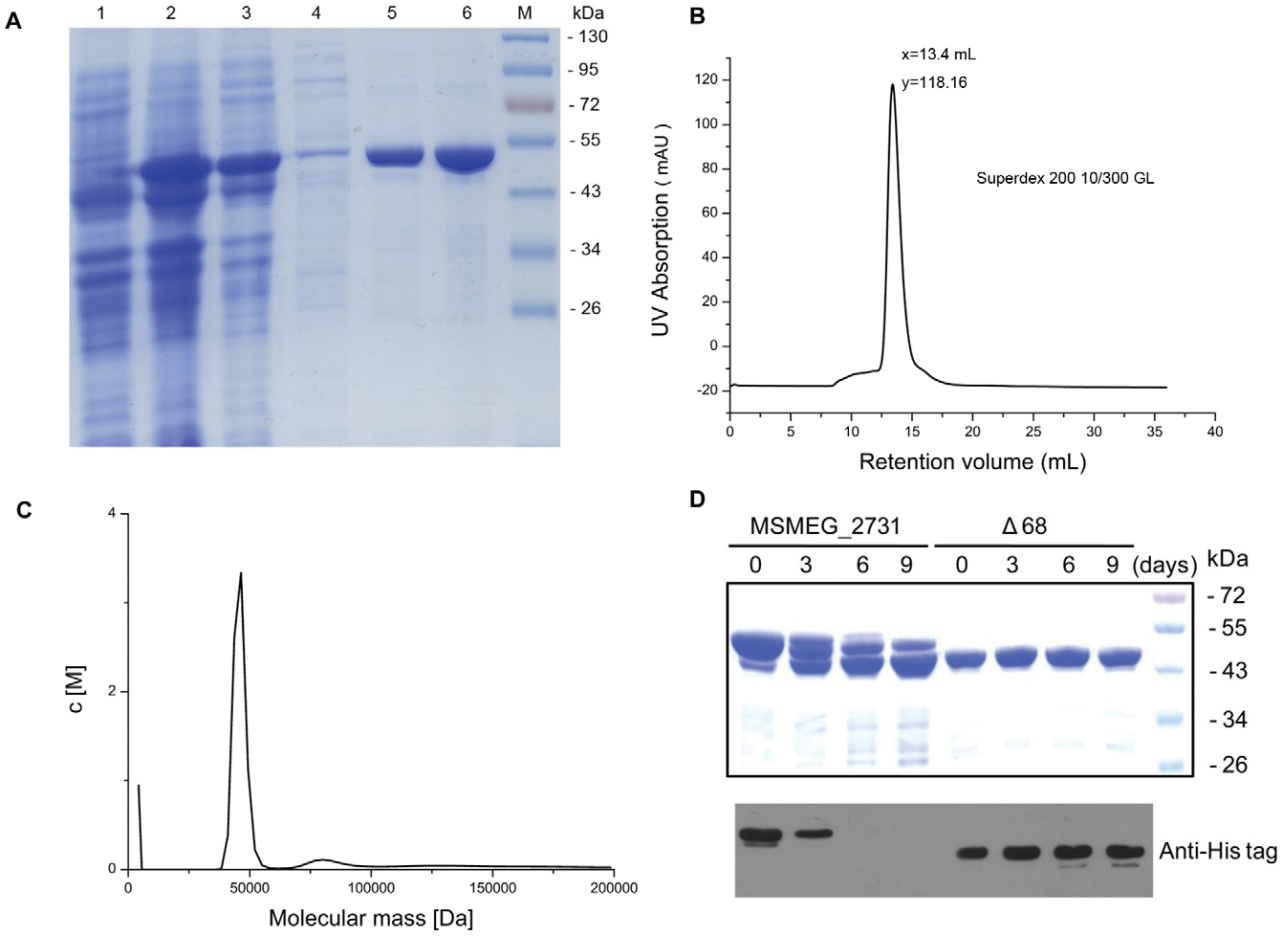
Purification and characterization of MSMEG_2731. (A) Purification of MSMEG_2731 by nickel affinity chromatography. Lane 1, BL21/pET28a-*MSMEG_2731* prior to IPTG induction, lane 2, BL21/pET28a-*MSMEG_2731* after IPTG induction, lane 3, the supernatant of the cell lysate after sonication, lane 4, sample after washing with buffer containing 30 mM imidazole, lane 5 and 6, two consecutive elutions with 250 mM imidazole. (B) A280 profile from gel filtration chromatography of recombinant MSMEG_2731 on a Superdex 200 10/300 column. The peak fraction was collected for analytical ultracentrifugation analysis. (C) Sedimentation velocity was determined on a Beckman Optima XL-I analytical ultracentrifuge at 20°C. The protein sample concentration was 0.8 mg/ml. Sedfit program was used to analyze the data. (D) Coomassie-stained gel and Western blotting showing the difference in stability between full-length MSMEG_2731 and Δ68 over time. Newly purified protein samples were stored at 4°C. Aliquots of protein were removed from the tube on days 3, 6 and 9 and mixed with the same volume of 2 x loading buffer. After boiling for 5 min and centrifugation, the samples were frozen at −70°C. Coomassie blue staining and Western blotting using an anti-His tagged antibody were utilized respectively to detect the degradation of MSMEG_2731 and Δ68 after SDS-PAGE.

MSMEG_2731 was expressed in *Escherichia coli* and the N-terminal His-tagged recombinant protein was purified with nickel affinity chromatography (Figure 1A). We used gel filtration (Figure 1B), to purify the target protein to homogeneity. Ultracentrifugation was used to test for oligomerization of MSMEG_2731 in solution. Sedimentation velocity experiments revealed that MSMEG_2731 has a molecular mass of 46 kDa (Figure 1C). As the theoretical molecular weight is 51.76 kDa, we concluded that MSMEG_2731 exists mostly as a monomer in solution. Except the main peak in sedimentation velocity profile that represented the monomers of MSMEG_2731, we also found a small peak that represented the dimers of MSMEG_2731. This result was verified by cross-linking assay (Figure S1).

**Figure 2:**
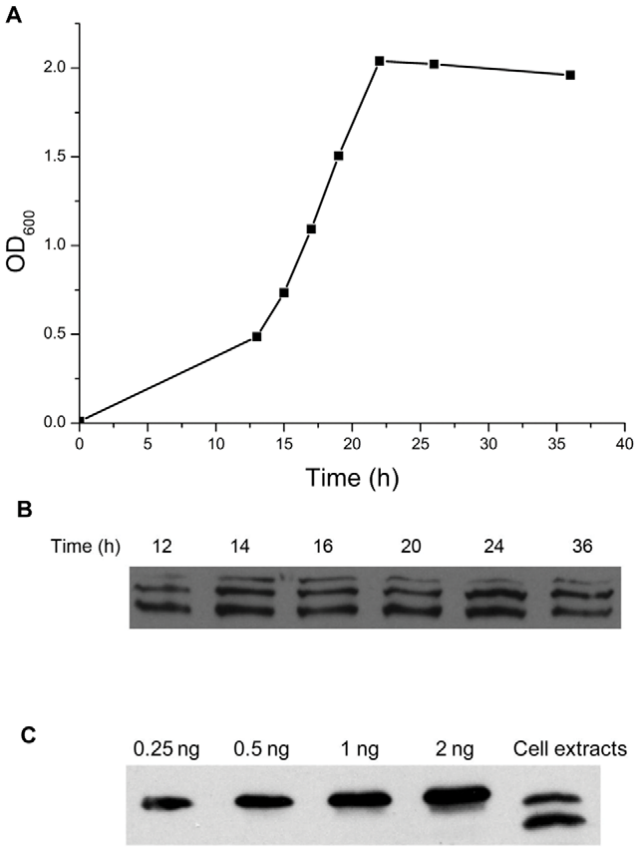
Identification of MSMEG_2731 *in vivo*. (A) The growth curve of *M. smegmatis*. A 1% inoculum of bacteria, freshly grown to an OD_600_≈2.0 was collected and washed with fresh 7H9 broth. It was then added to fresh 7H9 broth to give an OD_600_≈0.01 and further incubated with shaking (150 rpm) at 37°C for 48 h. The absorbance of the culture was monitored with a spectrophotometer at given intervals. (B) Cells harvested after 12 h, 14 h, 16 h, 20 h, 24 h and 36 h of incubation were resuspended in fresh 7H9 broth to give an OD_600_≈1.0. Then, 1 ml of the culture suspension was centrifuged to collect the cells. The pellets were resuspended in 40 μl of Laemmli sample buffer and boiled at 100°C for 10 min. Samples were electrophoresed on an SDS-PAGE gel and then transferred to a nitrocellulose membrane. Anti-MSMEG_2731 serum was utilized to detect the expression level of MSMEG_2731. (C) Estimation of the cellular concentration of MSMEG_2731 by quantitative Western blotting. Lanes 1 to 4 are recombinant MSMEG_2731 loaded at the indicated amounts. Lane 5 shows native MSMEG_2731 in exponentially-growing *M. smegmatis* cells (OD_600_ = 0.3).

### MSMEG_2731 tends to degrade at its N-terminus *in vitro*

We noted a band of a slightly lower molecular weight than MSMEG_2731 on SDS-PAGE gels. Using MALDI-TOF MS, we showed that this band isMSMEG_2731, but is probably a truncated form due to degradation of either or both of its terminals. We predicted the secondary structure of MSMEG_2731 using the PSIPRED protein structure prediction server (http://bioinf.cs.ucl.ac.uk/psipred/), and found that MSMEG_2731 is mostly composed of α helical motifs, except for 68 amino acids at the N-terminus which are disordered (Figure S2). We then constructed an N-terminal 68-aa deletion mutant and compared its stability with that of full-length MSMEG_2731. Coomassie blue staining demonstrated that MSMEG_2731 degraded markedly over time, whereas mutant protein Δ68 remained intact. Additionally, Western blotting showed that the His tag on the N terminus of MSMEG_2731 disappeared quickly and cannot be detected in 6 days, while the His tag on N-terminus of Δ68 was stable over time (Figure 1D). These results show that MSMEG_2731 tends to degrade at its N-terminus *in vitro*.

**Figure 3:**
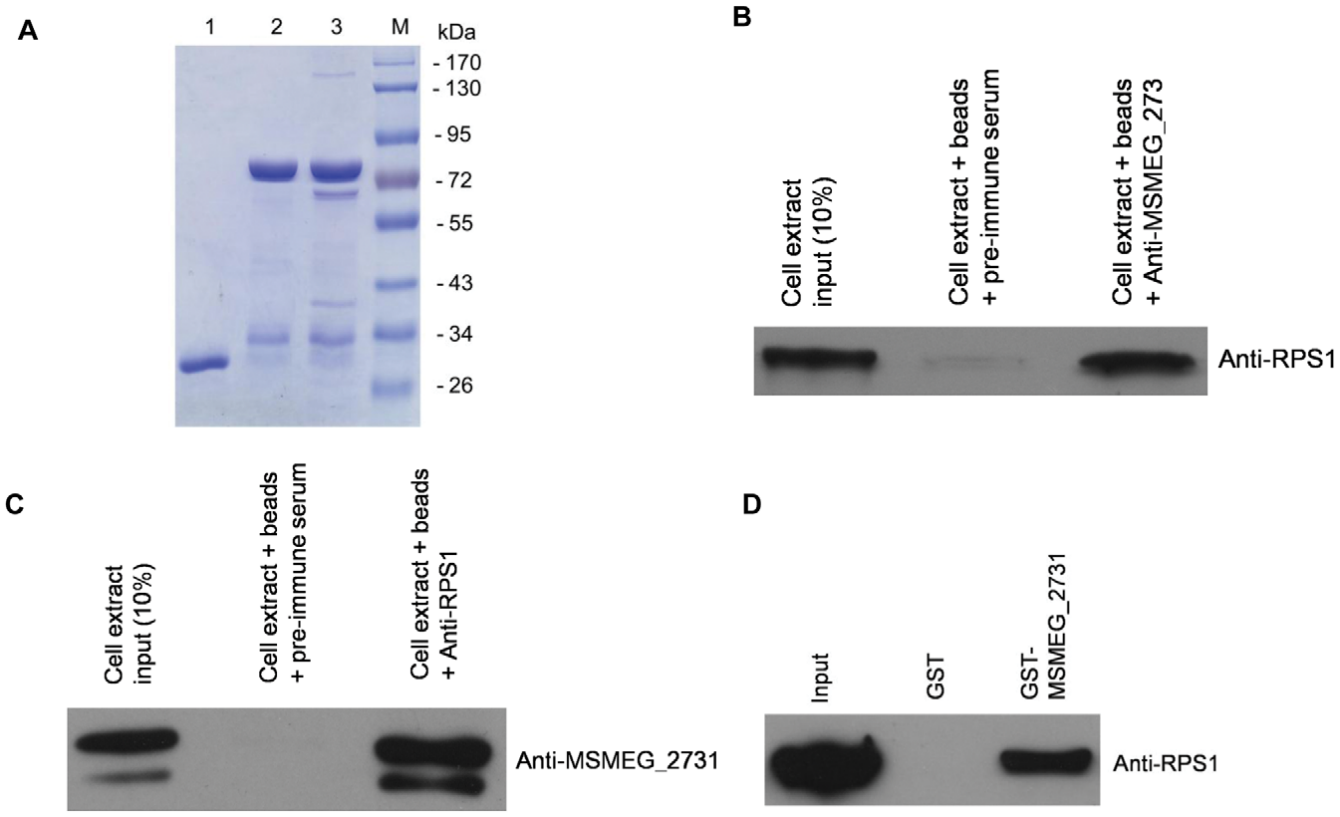
Isolation of MSMEG_2731 binding proteins by GST pull-down assays. (A) GST pull-down assay were performed as described in the materials and methods. Lane 1, GST incubated with cell lysates; lane 2, GST-MSMEG_2731 incubated with PBS; lane 3, GST-MSMEG_2731 incubated with cell lysates; lane 4, protein molecular markers. Several significant bands present in lane 3 but not in lane 1 and 2 were excised for identification by LC-MS/MS. (B) Verification of the interaction between MSMEG_2731 and RPS1. Endogenous RPS1 was immunoprecipitated with anti-MSMEG_2731 serum from *M. smegmatis* cell extracts, but not with pre-immune antiserum. The immunoprecipitated proteins were detected with anti-RPS1 serum. Ten percent of the cell extract was loaded as a control. (C) Endogenous MSMEG_2731 was immunoprecipitated with anti-RPS1 serum from *M. semegmatis* cell extracts, but not with pre-immune antiserum. (D) GST pull-down assay for examining the interaction between MSMEG_2731 and RPS1. Equimolar amounts of RPS1 combined with GST-MSMEG_2731 were used for the pull-down assay. GST was used as a negative control. The mixture was incubated for 1 h at 16°C, and then purified using a GST affinity assay. Samples were examined by Western blotting. RPS1 was pulled down by GST-MSMEG_2731, but not by GST.

**Table 1:** Peptides of ribosomal protein S1 identified by mass spectrometry.

Protein	Peptide
	R.THAIGQIVPGK.V
	K.FAAAEAEAANAPVSNGSSR.S
	R.VRDLQPYIGK.E
	R.RAWLEQTQSEVR.S
30S ribosomal	K.QANEDYTEEFDPSK.Y
protein S1	R.AWLEQTQSEVR.S
[Mycobacterium	R.SEFLNQLQK.G
smegmatis str.	R.AWGTIEELKEKDEAVK.G
MC2 155]	R.HVEVPDQVVQVGDDAM*VK.V
	R.HVEVPDQVVQVGDDAMVK.V
	R.SEESSGGTLASDAQLAALR.E
	K.VDRDEVLLDIGYK.T
	K.YFNDGDIVEGTIVK.V
	R.GFLPASLVEM*R.R
	R.VEEGIEGLVHISELSER.H
	K.GGLILDIGLR.G
	R.GFLPASLVEMR.R
	K.HDVDPNEVVSVGDEVEALVLTK.E

**Figure 4:**
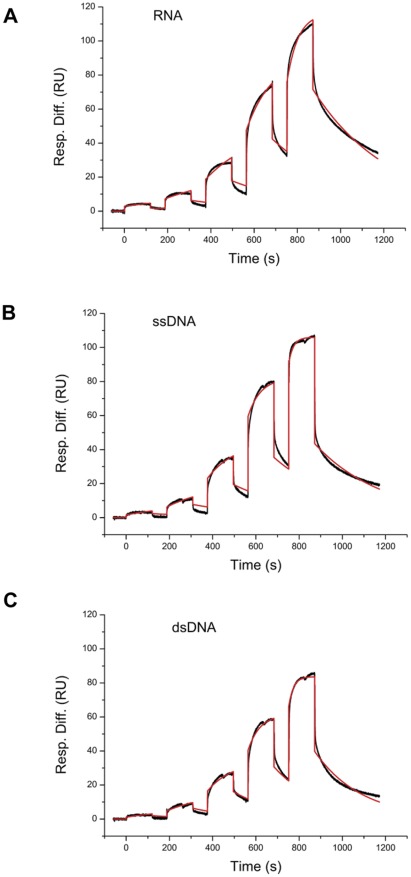
Kinetic profile of MSMEG_2731 binding to RNA, ssDNA and dsDNA. Sensorgrams generated using a Biacore T100 with a kinetic fit 1∶1 interaction model. Black lines represent the experimental data, and red lines represent the kinetic fit. A series of concentrations of MSMEG_2731 (0.2–16.2 μM) was injected in a single-cycle without regenerating the surface between injections. The association was monitored for 2 min, and the final dissociation time was 5 min. RNA was immobilized on a SA chip at 20 RU, and ssDNA and dsDNA were immobilized at 30 RU and 60 RU respectively. (A) Kinetic profile of MSMEG_2731 with RNA. (B) Kinetic profile of MSMEG_2731 with ssDNA. (C) Kinetic profile of MSMEG_2731 with dsDNA. The kinetic parameters are shown in Table 2.

**Table 2:** Kinetic parameters of interactions between MSMEG_2731 and RNA, ssDNA and dsDNA.

	Ka(M^−1^S^−1^)	Kd(S^−1^)	K_D_(M)
MSMEG_2731 and RNA	1027	2.82×10^−3^	2.746×10^−6^
MSMEG_2731 and ssDNA	2615	3.19×10^−3^	1.218×10^−6^
MSMEG_2731 and dsDNA	2506	4.61×10^−3^	1.840×10^−6^

**Figure 5:**
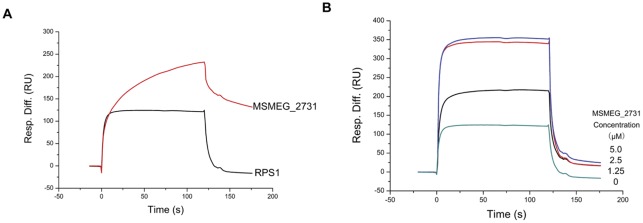
The MSMEG_2731-RPS1 complex interacts with RNA. (A) RNA was immobilized on a SA chip at 100 RU in running buffer containing 25 mM Tris-HCl (pH 7.4), 150 mM NaCl and 0.005% (v/v) Tween 20. RPS1 (2.5 μM) and MSMEG_2731 (2.5 μM) were successively passed over the immobilized RNA. The association was monitored for 2 min. RPS1 bound to RNA more quickly, and dissociated from RNA more completely than MSMEG_2731, while MSMEG_2731 bound to RNA with a higher RU. (B) RPS1 and MSMEG_2731 were mixed together at different ratios to obtain the following mixtures, 2.5 μM RPS1 and 1.25 μM MSMEG_2731, 2.5 μM RPS1 and 2.5 μM MSMEG_2731, and 2.5 μM RPS1 and 5 μM MSMEG_2731. The mixtures were successively passed over the immobilized RNA. Association and dissociation were monitored on a Biacore T100. The kinetic profile of the mixture binding to RNA was similar to that of RPS1. The presence of MSMEG_2731 led to a marked rise in the strength of binding, but 5 μM MSMEG_2731 had the same effect as 2.5 μM of MSMEG_2731.

**Table 3:** Oligonucleotides used in SPR assay.

oligonucleotides	sequence
dsDNA	5′-biotin-CACGGAGGACGAGGAGCGGAGATGA-3′
	3′ GTGCCTCCTGCTCCTCGCCTCTACT-5′
ssDNA	5′-biotin-CACGGAGGACGAGGAGCGGAGATGA-3′
RNA	5′-biotin-CACGUAUCUCGAGGAGCGGAUAUGA-3′

### Cellular concentration of MSMEG_2731 in *M. smegmatis*

To confirm the existence and measure the expression level of MSMEG_2731 *in vivo*, we analyzed samples taken from various stages of the growth cycle of *M. smegmatis* using Western blotting. Two bands were detected by the antiserum in cultures which is in the early log phase, and three bands were detected after the log phase. These bands represented different fragments of MSMEG_2731 due to its degradation *in vivo*. Nevertheless, we compared their amounts and concluded that MSMEG_2731 exists at a relatively stable level throughout the growth cycle (Figure 2A, B). We then estimated the cellular concentration of MSMEG_2731 using quantitative Western blotting (Figure 2C). The amount of native MSMEG_2731 was estimated from densitometric comparisons against recombinant MSMEG_2731 of known amounts. The number of cells was estimated using the drop plate method. Results from a semi-quantitative determination of cell counts show that the number of exponentially growing *M. smegmatis* cells at an OD_600_ of 0.3 is 4.5×10^7^. The cellular concentration of MSMEG_2731 was calculated using this data. Our results indicate that there are about 700 MSMEG_2731 molecules in each exponentially growing *M. smegmatis* cell.

### Isolation of MSMEG_2731 binding proteins

To elucidate the function of MSMEG_2731, we searched for cellular interaction partners by performing a GST pull-down assay using GST-tagged MSMEG_2731. Highly purified GST-MSMEG_2731 was bound to glutathione-Sepharose 4B beads and then incubated with cleared *M. smegmatis* cell lysates. Incubations of GST with cell lysates or GST-tagged MSMEG_2731 with PBS were utilized as negative controls. Proteins retained with the beads after extensive washing were analyzed by Coomassie-stained SDS-PAGE gels (Figure 3A). Several significant bands were excised and digested by trypsin. Subsequent identification of these samples by LC-MS/MS revealed that the interaction partners of MSMEG_2731 include the 30S ribosomal protein S1 (RPS1) (Table 1), DNA or RNA helicase of superfamily protein II (XPB), DNA-directed RNA polymerase subunit beta' (RpoC) and translation initiation factor IF-2 (Table S1, Excel S1). This result suggests that MSMEG_2731 may associate with proteins involved in translation or transcription.

### Verification of the interaction between MSMEG_2731 and RPS1

Co-immunoprecipitation was performed to verify the interaction between MSMEG_2731 and RPS1 *in vivo*. MSMEG_2731 and RPS1 immunoprecipitated together (Figure 3B, 3C) with both MSMEG_2731 antiserum and RPS1 antiserum, but not with rabbit pre-immune serum or rat pre-immune serum, confirming that MSMEG_2731 is associated with RPS1 physiologically. To clarify whether the interaction is direct or mediated by other factors, GST-tagged MSMEG_2731 was immobilized on glutathione-Sepharose 4B beads and used to pull down purified RPS1. RPS1 binds strongly to GST-MSMEG_2731, but not to GST alone (Figure 3D). This result verifies that MSMEG_2731 interacts directly with RPS1 *in vitro*.

### MSMEG_2731 is a nucleic acid binding protein

The interaction partners of MSMEG_2731 identified by MS prompted us to question whether MSMEG_2731 can bind to nucleic acids. In order to test our hypothesis, surface plasmon resonance (SPR) assays were performed to study the interaction between MSMEG_2731 and nucleic acids. 5′ biotinylated RNA, ssDNA and dsDNA were immobilized in three different channels on a SA chip at 20 resonance units (RU), 30 RU and 60 RU, respectively. A series of solutions containing different concentrations of MSMEG_2731 was passed through the three channels simultaneously. The response curves were recorded and analyzed using single-cycle kinetic methods on a BIAcore T100 machine. Interactions between MSMEG_2731 and RNA, ssDNA and dsDNA fit well with the simple 1∶1 model (Figure 4A, 4B, and 4C). Kinetic parameters shown in Table 2 show that the MSMEG_2731-RNA interaction has the lowest affinity and slowest association rate.

### Interaction between the MSMEG_2731-RPS1 complex and RNA

To determine whether MSMEG_2731 affects the binding of RPS1 with RNA, we compared the binding profile of RPS1 and RNA with that of RPS1, RNA and MSMEG_2731 on a Biacore T100 machine. RPS1 bound to RNA quickly and dissociated from RNA completely after injection. MSMEG_2731 bound to RNA at a lower rate and did not dissociate from RNA as completely as RPS1 (Figure 5A). Then we tested how MSMEG_2731-RPS1 complex interacted with RNA using the same chip. Just as RPS1 did, the complex bound to RNA rapidly and disassociated from RNA completely (Figure 5B). We conclude that RNA mainly binds with RPS1 and that the interaction between RPS1 and MSMEG_2731 does not affect RPS1 binding to RNA. To determine the binding ratio of MSMEG_2731:RPS1, we tested the binding strength of different amounts of MSMEG_2731 with fixed amount of RPS1. Maximum binding strength was achieved when the molar ratio was 1∶1, indicating that the interaction sites on RPS1 were totally occupied by MSMEG_2731. Thereafter, increasing the concentration of MSMEG_2731 to 2-fold of RPS1 cannot improve binding strength. Thus, it can be concluded that the binding ratio of MSMEG_2731:RPS1 is 1∶1.

## Discussion

MSMEG_2731 and its homologous proteins are highly conserved hypothetical proteins in mycobacteria (Figure S3). In the present study, we demonstrated the presence of MSMEG_2731 in *M. smegmatis* for the first time and explored its function by elucidating its biochemical characteristics and interaction partners. MSMEG_2731 is expressed at a stable level throughout the growth cycle. The expression of this conserved hypothetical gene implies that it may play a role in mycobacterial specific functions.

Protein secondary structure prediction shows that MSMEG_2731 is mostly composed of alpha helices, except for 68 disordered N-terminal amino acids. Disordered protein regions are known to play a role in many vital biological processes, such as cell signaling, recognition, and nucleic acid and protein-protein interactions [11], [12]. By analyzing its composition of amino acids, we found that this unstructured region contains 25% proline and 13.2% arginine, and that its theoretical pI is 11.70, significantly higher than that of the full-length protein (pI = 7.07). This implies that these 68 N-terminal amino acids may mediate the interaction of MSMEG_2731 with nucleic acids or other proteins. However, our results showed that MSMEG_2731 tends to degrade at it N-terminus *in vitro*. In addition, the detection of two or three bands in *M. smegmatis* cell lysates using anti-MSMEG_2731 serum indicates that the N-terminus of MSMEG_2731 may also be unstable *in vivo*. Nevertheless, loss of these 68 N-terminal amino acids does not affect the interaction of MSMEG_2731 with nucleotides or RPS1 (data not shown). Meanwhile, the N-terminus is the least conserved region in MSMEG_2731 and its homologous proteins in mycobacteria. Thus further study is required to clarify whether the most N-terminal amino acids are indispensable in MSMEG_2731 or not.

Ribosomal protein S1 (RPS1) is found in all Gram-negative and in several Gram-positive bacteria [13]. RPS1 is the largest ribosomal protein and is located at the junction of the head, platform, and main body of the 30S subunit [14]. Ribosome preparations often contain less than stoichiometric amounts of RPS1, implying that the association of RPS1 with the ribosome is weak and reversible compared with other ribosomal proteins [15]. It has been suggested that RPS1 is necessary for translation initiation and translation elongation [16], [17]. RPS1 has also been reported to interact with RNA polymerase and to promote transcriptional cycling *in vitro* [18], [19]. All these facts suggest that RPS1 functions at the interface of transcription and translation. *E. coli* RPS1 consists of six similar domains that play different roles. The first two domains are involved in binding to the ribosome, while the last four are involved in interactions with mRNA [20], [21]. Four S1 domains have been identified in mycobacterial RPS1, corresponding to the first four domains of its counterpart in *E. coli* [22]. Yamada suggested that RPS1 from *M. smegmatis* is functionally similar to *E. coli* RPS1 with respect to stimulating poly(U)-directed polyphenylalanine synthesis [23]. Using surface plasmon resonance, we show that *M. smegmatis* RPS1 can bind to RNA quickly and dissociates from RNA completely, which may be relevant to its function *in vivo*.

RPS1 was identified by Shi *et* *al* along with three other proteins Rv2731, Rv2783c and Rv3169 in a search for the target of pyrazinoic acid (POA) in *M. tuberculosis* [24]. It is possible that Rv2731 was pulled-down in this case due to its association with RPS1. Based on our findings that MSMEG_2731 can bind to RNA and interacts with RPS1, we hypothesize that MSMEG_2731 may associate with RPS1 and regulate its function involved in transcription and translation. Although MSMEG_2731 does not affect the binding of RPS1 to RNA, it may compete with other proteins, such as ribosomal proteins and RNA polymerase, for the protein-protein interaction interface on RPS1.

In addition to RPS1, DNA or RNA helicase of superfamily protein II (XPB), DNA-directed RNA polymerase beta' subunit (RpoC) and translation initiation factor IF-2 were identified as direct or indirect interaction partners of MSMEG_2731 (Table S1). XPB is the largest subunit of the eukaryotic TFIIH complex which plays an important role in both the initiation of transcription and nucleotide excision repair [25], [26]. XPB is almost exclusively present in eukaryotes, however genes highly homologous to human XPB have been found in mycobacteria [27]. The interactions of MSMEG_2731 with XPB, RpoC or IF-2 observed here make our hypothesis that MSMEG_2731 may be involved in transcription and translation more convincing.

Although MSMEG_2731 is annotated as an ATPase involved in DNA repair, there are no common ATP binding domains on MSMEG_2731. Actually, we did not detect its ATPase activity *in vitro* (data not shown). Besides, we construct MSMEG_2731 knocked-out mutants (Figure S4), and find that the spontaneous mutation rate of the *MSMEG_2731* knocked-out mutant is almost equal to that of the wild type (data not shown). Therefore, MSMEG_2731 appears to not be a DNA repair protein. Our results that MSMEG_2731 binds to RNA and interacts with RPS1 physically and physiologically suggests it play a role in transcription or translation process. However, more extensive investigation into the function of MSMEG_2731 is required to clarify whether this conserved hypothetical protein carry out its function in mycobacterial specific regulation of transcription or translation.

## Materials and Methods

### Plasmid construction

All primers sequences used are listed in Table S2. Genomic DNA from *M. smegmatis* was isolated using an E.Z.N.A.® Bacterial DNA Kit from Omega Bio-Tek Inc. Genes encoding RPS1, MSMEG_2731 and its truncated forms were amplified by PCR using genomic DNA from *M. smegmatis* as the template. DNA fragments were digested by restriction enzymes and ligated into pET28a or pGEX-6P-1 vectors. The ligated plasmid constructs were introduced into *E. coli* strain BL21 (DE3), and the sequences of the cloned DNA fragments were confirmed by DNA sequencing.

### Antibody production

Purified recombinant MSMEG_2731 was used to immunize rabbits to obtain MSMEG_2731 antiserum. RPS1 antiserum was obtained by immunizing rats with purified recombinant RPS1.

### Protein expression and purification

All proteins were expressed in *E. coli* BL21 (DE3) cells in Luria-Bertani (LB) medium. Cells were cultured at 37°C, (shaken at 220 rpm) to an OD_600_ ∼0.4 and induced with isopropyl thio β-D-galactoside (final concentration 0.4 mM) at 16°C overnight. Cells were harvested by centrifugation at 5000 rpm for 30 min and then lysed by sonication. Cell fragments were separated by centrifugation at 16,000 rpm for 30 min at 4°C. His-tagged proteins were purified using a Ni^2+^-NTA column (Amersham) and gel filtration, while GST-tagged proteins were purified using glutathione-Sepharose 4B beads (Amersham). The purity of proteins was determined by sodium dodecyl sulfate-polyacrylamide gel electrophoresis (SDS-PAGE). After the concentration of each protein was measured by UV absorption at A280, proteins were divided into aliquots, frozen in liquid nitrogen and stored at −80°C.

### Quantitative Western blotting

Quantitative Western blotting was performed according to the method of Mukherjee et al., with some modifications [28]. To detect the expression level of MSMEG_2731, 1 ml of *M. smegmatis* cells was harvested when the OD_600_ reached 0.3. Cell pellets were resuspended in 50 μl Laemmli sample buffer and then boiled at 90°C for 30 min. Samples were electrophoresed by 12% SDS-PAGE and transferred to a nitrocellulose membrane. The polyacrylamide gel was then stained with Coomassie blue to ensure complete transfer of proteins. The nitrocellulose membrane was blocked with 5% nonfat milk powder dissolved in 1× PBST for 1 h at room temperature and the membrane was then incubated overnight at 4°C with 1∶500 dilution of MSMEG_2731 antiserum. After washing the membrane three times with 1× PBST, it was incubated with goat anti-Rabbit immunoglobulin G horseradish peroxidase-conjugated secondary antibody at room temperature for 1 h. The membrane was then washed three times with 1× PBST and incubated with chemiluminescent substrates (SuperSignal® West Pico, Bio-Rad). After exposing the blot to Fuji film for 30 seconds, the film was scanned and then analyzed by Quantity One® software. The concentration of MSMEG_2731 was calculated as a function of the integrated density of the bands. The amount of MSMEG_2731 in the *M. smegmatis* sample was calculated against a recombinant MSMEG_2731 standard.

### Mass spectrometry

The purified recombinant GST-MSMEG_2731 was incubated with M. smegmatis cell lysates for 4 h in lysis buffer B (50 mM Tris-HCl (pH 7.4), 150 mM NaCl, 1 mM EDTA, 0.1% Triton X-100). A negative control using GST alone as a bait protein was run at the same time. A 50-μl slurry of glutathione-Sepharose 4B beads was added and incubated with the system for another 1 h. After centrifugation at 700× g, the supernatant was discarded, and the pellets were washed five times with 800 μl buffer B at 4°C. After washing, the pellets were resuspended in 20 μl of Laemmli sample buffer followed by boiling at 100°C for 10 min. The sample was centrifuged at 12 000 g for 5 min and then electrophoresed on a 12% SDS-PAGE gel. The major bands present in the sample but not in the control were excised and in-gel digested with trypsin. The peptides were then analyzed by LC-MS/MS on a ProteomeX-LTQ mass spectrometer (Thermo Fisher Scientific, Waltham, USA).

### Co-IP assay

Exponentially-growing cells of *M. smegmatis* were harvested, resuspended and lysed in lysis buffer B (50 mM Tris-HCl (pH 7.4), 150 mM NaCl, 1 mM EDTA, 0.1% Triton X-100). Cell extracts were centrifuged at 30,700 g for 30 min at 4°C. Supernatants were transferred and incubated with 5 μl of MSMEG_2731 antiserum for 4 h at 4°C. 20 μl of protein G agarose slurry was then added to the solution and incubated for 1 h. Beads were collected by centrifugation at 700 g for 5 min, then washed five times with lysis buffer at 4°C, and resuspended in SDS-PAGE sample loading buffer. The sample was electrophoresed on a 12% SDS-PAGE gel and then analyzed by Western blotting.

### SPR experiments

SPR experiments were carried out on a BIAcore T100 machine (GE Healthcare) at 25°C. To measure and compare the strength of the interactions of MSMEG_2731 with double-stranded DNA, single-stranded DNA and RNA, 5′ biotinylated dsDNA (25 bp), ssDNA (25 nt) and RNA (25 nt) were immobilized in different channels on a streptavidin (SA)-coated sensor chip (Table 3). The sensor chip was equilibrated with running buffer at a flow rate of 30 μl/min until the baseline was stable. The running buffer contained 25 mM Tris-HCl (pH 7.4), 150 mM NaCl and 0.005% (v/v) Tween 20. Protein samples were injected at different concentrations using a flow rate of 30 μl/min for 2 min. Kinetic analysis was performed using a single-cycle kinetics approach in all the SPR experiments.

## Supporting Information

Figure S1 **Cross-linking assay of MSMEG_2731 using BS3.** MSMEG_2731 with increasing concentrations (lanes 2–9; 0.025, 0.05, 0.1, 0.25, 0.5, 1, 2.5, 5, 10 mM, respectively) of BS3 were incubated in non-amine-containing buffer for 30 min at room temperature. The reaction was quenched by adding 1 M Tris-HCl to a final concentration of 50 mM Tris. The samples were resolved by 12% SDS-PAGE. Coomassie blue staining showed that BS3 induced several bands with larger molecular weight, which represented the dimers and other forms of oligomers of MSMEG_2731. But much excess of BS3 abrogated the cross-linked complex. This effect may be due to the reason that oversaturated BS3 left no available primary amines for two adjacent protein to bind each other.
(TIF)

Figure S2 **Schematic illustrating the modular composition of MSMEG_2731 and its mutants.** MSMEG_2731 is composed of three sections, the N-terminal domain (1–142), the three tandem repeats of DUF349 (143–373) and the C-terminal domain (374–454). The mutant constructed in this study is MSMEG_2731 (69–454) (Δ68).
(TIF)

Figure S3 **Multiple sequences alignment of MSMEG_2731 and its homologous proteins from other mycobacteria.** The sequences in the alignment are from (top to bottom): *Mycobacterium bovis, Mycobacterium tuberculosis, Mycobacterium smegmatis, Mycobacterium avium, Mycobacterium marinum*.
(TIF)

Figure S4 **Construction of the MSMEG_2731 deletion strains.** (A) The MSMEG_2731 gene was deleted from the *M. smegmatis* genome using the mycobacterial recombineering system. Briefly, the knockout cassette was generated by overlap extension PCR, in which the two 500 bp sequences fragments flanking each of the ends of MSMEG_2731 were fused with the hygR fragment. Then the knockout cassette was transformed into the wild type MC2 155 strains harboring the recombineering plasmid pJV53. The positive recombinants were identified by PCR analysis. (B) PCR results with primers flanking upstream homology. The 3′ primer is located in hygR sequence. ak1–9 are different strains selected from the plate. A wild-type strain was used as a control. (C) PCR results using hygR primers. A wild-type strain and hygR gene fragment are negative and positive controls respectively. (D) PCR results using primers flanking the downstream homology. The 5′ primer is located in the hygR sequence. A wild-type strain was used as negative control.
(TIF)

Table S1 **Peptides identified by mass spectrometry.**
(DOC)

Table S2 **PCR primers used in this study.**
(DOC)

Excel S1 **Mass Spectrometry results analysis.** All MS/MS data were searched against a *Mycobacterium smegmatis MC-155* protein database (2009-6-23, 16221 entries) downloaded from the NCBI database using the SEQUEST (v.28) program (Thermo, USA). All searches were performed using a precursor mass tolerance of 3 Da calculated using average isotopic masses. Variable modification was set for methionine with the addition of 15.999 Da to represent methionine oxidation; static modification was set for cysteine with the addition of 57.052 Da to represent cysteine carboxyamidation. A fragment ion mass tolerance of 1Da was used. Enzyme cleavage specificity was set to trypsin and no more than two missed cleavages were allowed. The SEQUEST outputs were then analyzed using the commercial software Thermo Electron BioWorks (Rev.3.3.1 sp1). The filter settings for peptides were as follows –distinct peptide, Xcorr ≥1.9 (z = 1), 2.7 (z = 2), 3.75 (z = 3), Sp≥500, Rsp≤5, at least two distinct peptides per protein.
(XLS)
