# Correction: MSMEG_2731, an Uncharacterized Nucleic Acid Binding Protein from *Mycobacterium smegmatis*, Physically Interacts with RPS1

**DOI:** 10.1371/annotation/ba9d097a-969c-4a2e-92a9-401929a6e934

**Published:** 2012-05-17

**Authors:** Mingzhang Yang, Yuanyuan Chen, Ying Zhou, Liwei Wang, Hongtai Zhang, Li-Jun Bi, Xian-En Zhang

There is an error in the following Author Contributions designation: Conceived and designed the experiments: MZY LJB XEZ. 

