# Supplementary figures and images for: MSMEG_2731, an Uncharacterized Nucleic Acid Binding Protein from Mycobacterium smegmatis, Physically Interacts with RPS1
Source: PLoS One. 2012 May 9;7(5):e36666. doi: 10.1371/journal.pone.0036666 (PMC3348880; doi:10.1371/journal.pone.0036666)

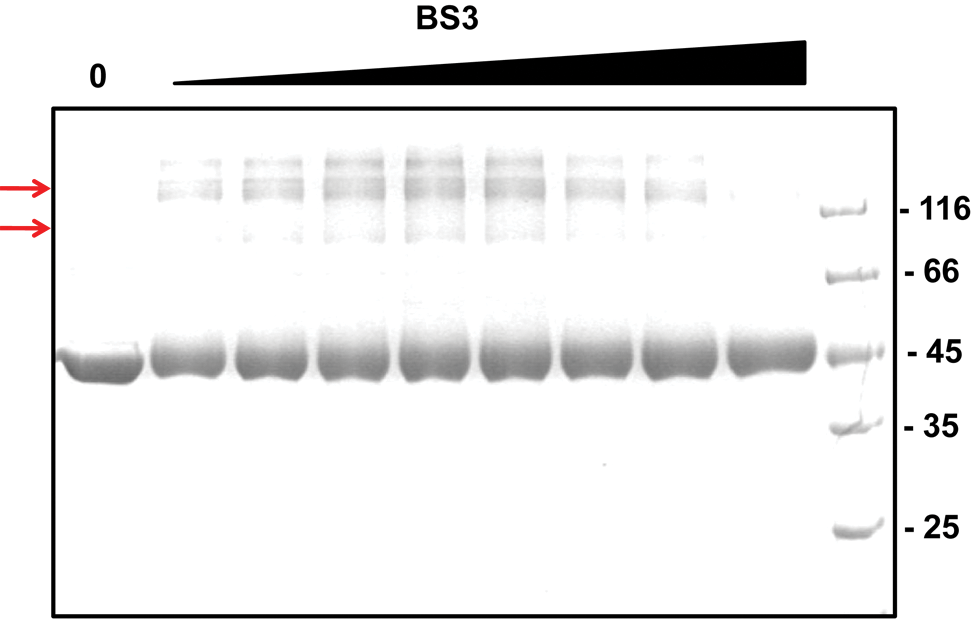

Supplement: Figure S1 — Cross-linking assay of MSMEG_2731 using BS3. MSMEG_2731 with increasing concentrations (lanes 2–9; 0.025, 0.05, 0.1, 0.25, 0.5, 1, 2.5, 5, 10 mM, respectively) of BS3 were incubated in non-amine-containing buffer for 30 min at room temperature. The reaction was quenched by adding 1 M Tris-HCl to a final concentration of 50 mM Tris. The samples were resolved by 12% SDS-PAGE. Coomassie blue staining showed that BS3 induced several bands with larger molecular weight, which represented the dimers and other forms of oligomers of MSMEG_2731. But much excess of BS3 abrogated the cross-linked complex. This effect may be due to the reason that oversaturated BS3 left no available primary amines for two adjacent protein to bind each other. (TIF) [file pone.0036666.s001.tif]

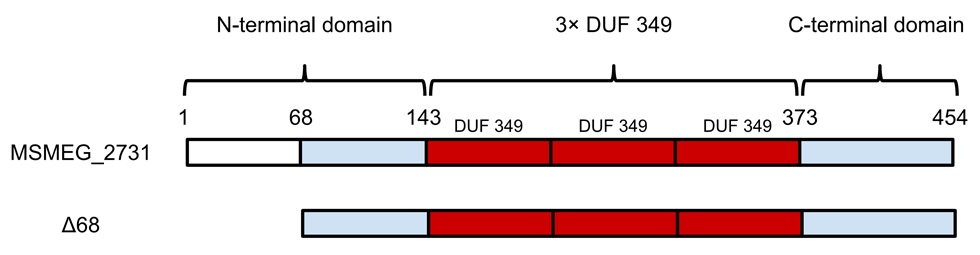

Supplement: Figure S2 — Schematic illustrating the modular composition of MSMEG_2731 and its mutants. MSMEG_2731 is composed of three sections, the N-terminal domain (1–142), the three tandem repeats of DUF349 (143–373) and the C-terminal domain (374–454). The mutant constructed in this study is MSMEG_2731 (69–454) (Δ68). (TIF) [file pone.0036666.s002.tif]

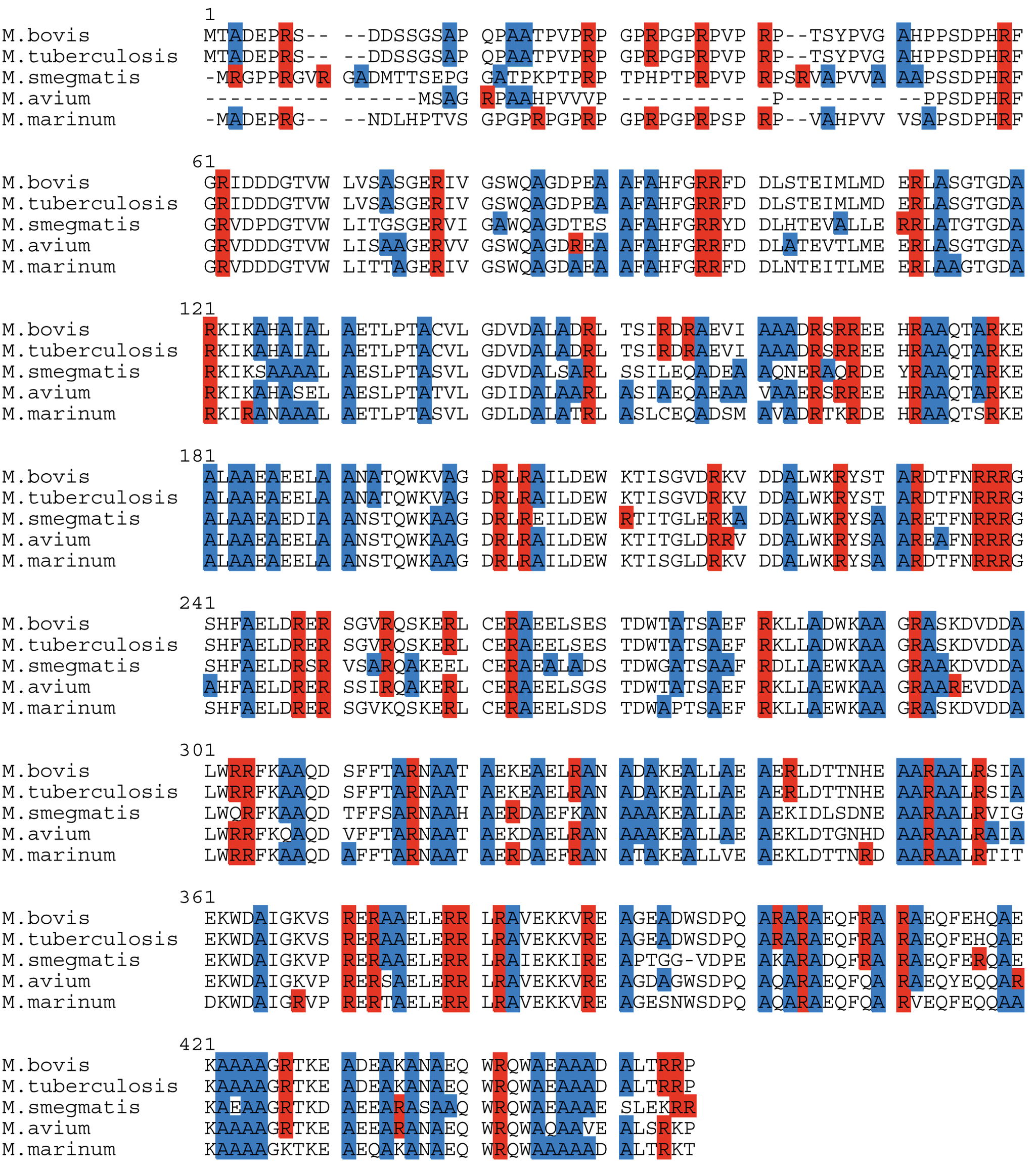

Supplement: Figure S3 — Multiple sequences alignment of MSMEG_2731 and its homologous proteins from other mycobacteria. The sequences in the alignment are from (top to bottom): Mycobacterium bovis, Mycobacterium tuberculosis, Mycobacterium smegmatis, Mycobacterium avium, Mycobacterium marinum. (TIF) [file pone.0036666.s003.tif]

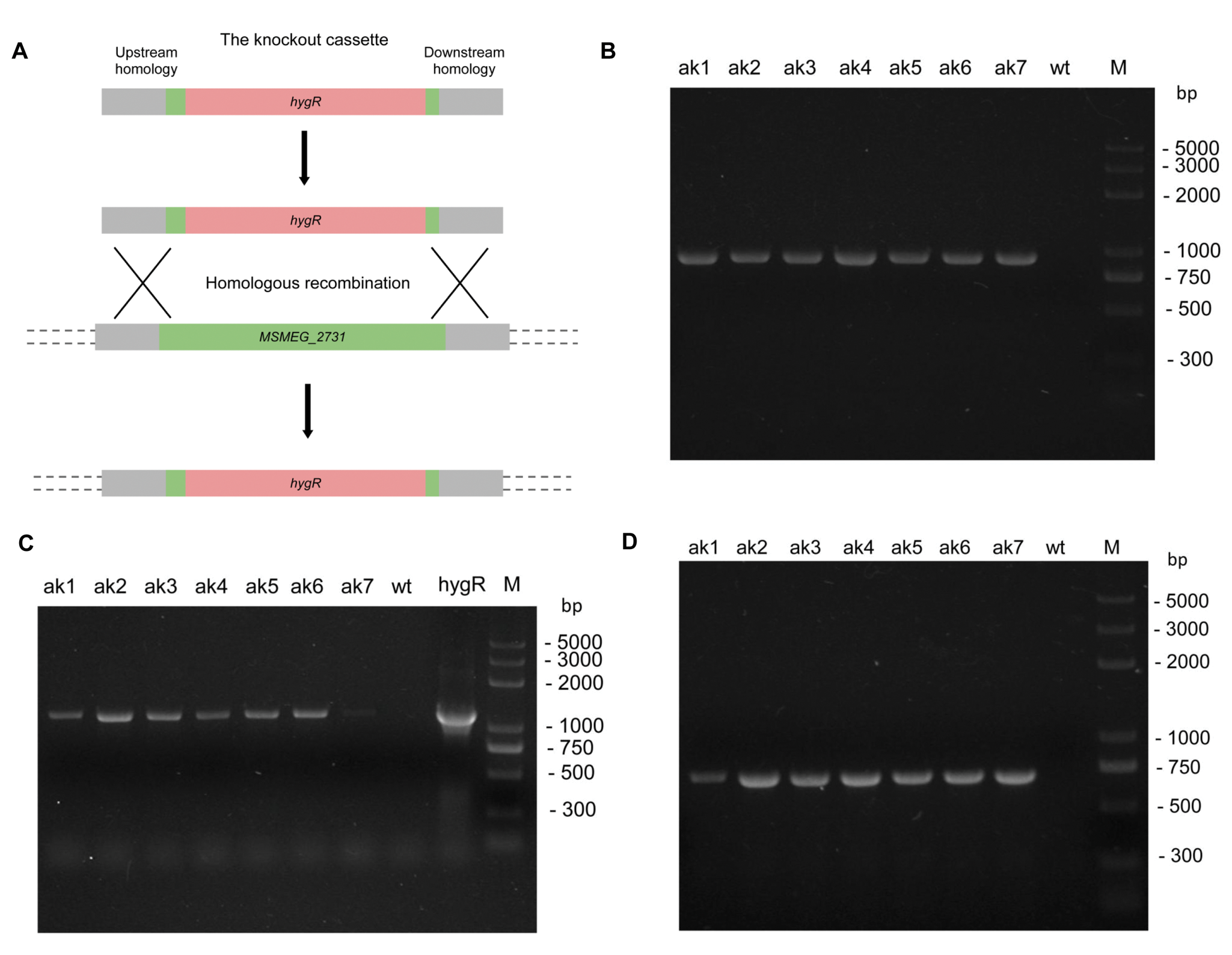

Supplement: Figure S4 — Construction of the MSMEG_2731 deletion strains. (A) The MSMEG_2731 gene was deleted from the M. smegmatis genome using the mycobacterial recombineering system. Briefly, the knockout cassette was generated by overlap extension PCR, in which the two 500 bp sequences fragments flanking each of the ends of MSMEG_2731 were fused with the hygR fragment. Then the knockout cassette was transformed into the wild type MC2 155 strains harboring the recombineering plasmid pJV53. The positive recombinants were identified by PCR analysis. (B) PCR results with primers flanking upstream homology. The 3′ primer is located in hygR sequence. ak1–9 are different strains selected from the plate. A wild-type strain was used as a control. (C) PCR results using hygR primers. A wild-type strain and hygR gene fragment are negative and positive controls respectively. (D) PCR results using primers flanking the downstream homology. The 5′ primer is located in the hygR sequence. A wild-type strain was used as negative control. (TIF) [file pone.0036666.s004.tif]
