# Supplementary material for: MSMEG_2731, an Uncharacterized Nucleic Acid Binding Protein from Mycobacterium smegmatis, Physically Interacts with RPS1
Source: PLoS One. 2012 May 9;7(5):e36666. doi: 10.1371/journal.pone.0036666 (PMC3348880; doi:10.1371/journal.pone.0036666)
Supplement: Table S1 — Peptides identified by mass spectrometry. (DOC) [file pone.0036666.s005.doc]

Table S1. Peptides identified by mass spectrometry

| Protein | Peptide |
| --- | --- |
|  | R.IDDDTVIVHNSER.G |
|  | R.TSLTEDEIGEYSGEKK.E |
| DNA or RNA helicase of | R.TSLTEDEIGEYSGEK.K |
| superfamily protein II | K.ADGGGAVFYSVVSR.D |
| [Mycobacterium | R.LGLTATLIR.E |
| smegmatis str. MC2 155] | K.TVLLEVDHELAGAAR.A |
|  | R.FLAEQGYGYIIK.D |
|  | K.AGATTLILVTNTVAGR.Q |
|  | R.ITPLALWNAR.A |
|  | R.AAGHDAEQVVDALVSFSR.Y |
|  | R.NIQVQPTEEAR.A |
| DNA-directed RNA | R.LVDLNHAQNIK.S |
| polymerase subunit beta' | R.LVDVSQDVIVR.E |
| [Mycobacterium | R.TLATDAVDANGNVIIER.G |
| smegmatis str. MC2 155] | K.LEADLAELEAEGAK.S |
|  | R.FPMIVVAQTVDK.L |
|  | R.LGIQAFEPQLVEGK.A |
| translation initiation factor | R.VGDSVVAGDAYGR.V |
| IF-2 [Mycobacterium | R.GPVATVLIQR.G |
| smegmatis str. MC2 155] | R.DNVVVAQNLTVSSLR.R |
|  | K.EGDVIETYELVEK.A |
