# Supplementary material for: MSMEG_2731, an Uncharacterized Nucleic Acid Binding Protein from Mycobacterium smegmatis, Physically Interacts with RPS1
Source: PLoS One. 2012 May 9;7(5):e36666. doi: 10.1371/journal.pone.0036666 (PMC3348880; doi:10.1371/journal.pone.0036666)
Supplement: Table S2 — PCR primers used in this study. (DOC) [file pone.0036666.s006.doc]

Table S2. PCR primers used in this study

| Plasmids | Primers | Restriction sites |
| --- | --- | --- |
| pET28a-*MSMEG_2731* | 5' -GGAATTCCATATGATGCGCGGGCCACC-3' | NdeI |
|  | 5'-CCCAAGCTTTCAGCGGCGCTTCTCCAGC-3' | HindIII |
| pQE30-*rpsA* | 5'-CGCGGATCCATGCCAAGTCCCTCCGTCACCTCG-3' | BamHI |
|  | 5'-TTCGAAGCTTCTAGGCGTTGCCGGCGAGCTTC-3' | HindIII |
| pGEX-6P-1- | 5'-CCGGAATTCATGCGCGGGCCACC-3' | EcoRI |
| *MSMEG_2731* | 5'-CCGCTCGAGTCAGCGGCGCTTCTCCAGC-3' | XhoI |
| pET28a-*MSMEG_2731* | 5'-GGAATTCCATATGTGGCTGATCACCGGATCC-3' | NdeI |
| (Δ68) | 5'-GGGCTCGAGTCAGCGGCGCTTCTCCA-3' | XhoI |
